# Impact of daily plan adaptation on accumulated doses in ultra-hypofractionated magnetic resonance-guided radiation therapy of prostate cancer

**DOI:** 10.1016/j.phro.2024.100562

**Published:** 2024-02-25

**Authors:** Yuqing Xiong, Moritz Rabe, Carolin Rippke, Maria Kawula, Lukas Nierer, Sebastian Klüter, Claus Belka, Maximilian Niyazi, Juliane Hörner-Rieber, Stefanie Corradini, Guillaume Landry, Christopher Kurz

**Affiliations:** a Department of Radiation Oncology, LMU University Hospital, LMU Munich, Munich, Germany; b Department of Radiation Oncology, Heidelberg University Hospital, Heidelberg, Germany; c Heidelberg Institute of Radiation Oncology, National Center for Radiation Oncology, Heidelberg, Germany; d German Cancer Consortium (DKTK), Partner site Munich, a Partnership between DKFZ and LMU University Hospital Munich, Germany; e Bavarian Cancer Research Center (BZKF), Munich, Germany; f Clinical Cooperation Unit Radiation Oncology, German Cancer Research Center, Heidelberg, Germany; g National Center for Tumor Diseases, Heidelberg, Germany; h German Cancer Consortium (DKTK), Heidelberg, Germany

**Keywords:** MRgRT, Dose accumulation, Prostate, Online plan adaptation, Ultra-hypofractionation

## Abstract

**Background and purpose:**

Ultra-hypofractionated online adaptive magnetic resonance-guided radiotherapy (MRgRT) is promising for prostate cancer. However, the impact of online adaptation on target coverage and organ-at-risk (OAR) sparing at the level of accumulated dose has not yet been reported. Using deformable image registration (DIR)-based accumulation, we compared the delivered adapted dose with the simulated non-adapted dose.

**Materials and methods:**

Twenty-three prostate cancer patients treated at two clinics with 0.35 T magnetic resonance-guided linear accelerator (MR-linac) following the same treatment protocol (5 × 7.5 Gy with urethral sparing and daily adaptation) were included. The fraction MR images were deformably registered to the planning MR image. Both non-adapted and adapted fraction doses were accumulated with the corresponding vector fields. Two DIR approaches were implemented. PTV* (planning target volume minus urethra^+2mm^) *D*_95%,_ CTV* (clinical target volume minus urethra) *D*_98%,_ and OARs (urethra^+2mm^, bladder, and rectum) *D*_0.2cc,_ were evaluated. Statistical significance was inferred from a two-tailed Wilcoxon signed-rank test (*p* < 0.05).

**Results:**

Normalized to the baseline, the accumulated PTV* *D*_95%_ increased significantly by 2.7 % ([1.5, 4.3]%) through adaptation, and the CTV* *D*_98%_ by 1.2 % ([0.1, 1.7]%). For the OARs after adaptation, accumulated bladder *D*_0.2cc_ decreased by 0.4 % ([−1.2, 0.4]%), urethra^+2mm^*D*_0.2cc_ by 0.8 % ([−1.6, −0.1]%), while rectum *D*_0.2cc_ increased by 2.6 % ([1.2, 4.9]%). For all patients, rectum *D*_0.2cc_ was still below the clinical constraint. Results of both DIR approaches differed on average by less than 0.2 %.

**Conclusions:**

Online adaptation in MRgRT improved target coverage and OARs sparing at the level of accumulated dose.

## Highlights

• First comparison of accumulated online adapted versus non-adapted doses for prostate magnetic resonance-guided radiation therapy was conducted.
• On average, limited improvements in target coverage and organs-at-risk sparing were observed.
• Online adaptation played a crucial role for patients with strong anatomic variations.

## 1 Introduction

Since the introduction of integrated magnetic resonance imaging (MRI) guided linear accelerators (MR-linacs) to the clinic, magnetic resonance-guided radiotherapy (MRgRT) has emerged as a novel approach for prostate cancer treatment [1], [2]. MRI is advantageous over cone beam computed tomography (CBCT) as an image-guidance technology for radiotherapy due to its dose-free nature and enhanced soft tissue contrast, which allows tumor localization without any external surrogates or fiducial markers [3], [4], [5]. This improves the accuracy of patient positioning and anatomical structure delineation [6]. MR-linacs also allow daily online adaptation of treatment plans to account for inter-fractional anatomical changes observed on the in-room set-up MRI scans [2], [7], [8], [9], [10]. Additionally, time resolved 2D imaging (cine MRI) can be acquired simultaneously with the beam delivery and used to gate the irradiation, enabling further margin reduction [11], [12]. In recent years, with increased evidence of the low *α/β* ratio of prostate cancer, and the availability of online adaptive MRgRT, there has been a growing use of ultra-hypofractionation as an alternative to conventional fractionation schemes [13]. Studies have demonstrated that ultra-hypofractionated radiotherapy can achieve similar clinical outcomes in terms of failure-free survival and late toxicity as conventional fractionated schemes, such as 39 × 2 Gy [14], [15], [16]. The enhanced soft tissue contrast of MRI has also brought a renewed focus on urethra dose sparing with avoidance of hotspots [17].

Previous studies have assessed the impact of the intra-fractional prostate motion captured during gated MRgRT on the delivered dose [18], [19], [20], [21]. The findings indicated that the magnitude of prostate intra-fractional motion is small, so that gating has a negligible effect on the delivered dose in most cases. Moreover, the benefit of online plan adaptation for prostate cancer patients has been examined retrospectively by comparing dose volume histogram (DVH) parameters of non-adapted and adapted doses at individual fraction level. No significant change in bladder and rectum exposure was observed on average, and planning target volume (PTV) and clinical target volume (CTV) coverage was mostly clinically acceptable even before adaptation [22]. However, an assessment of the consistency between the accumulated adapted dose over all treatment fractions and the baseline dose for ultra-hypofractionated prostate MRgRT has been lacking. To date, no investigation has been conducted to compare the accumulated online adapted dose with the accumulated dose that would be obtained in the absence of plan adaptation. The accumulated dosimetric advantage of performing the time-consuming online adaptation has not yet been quantified [23], [24], [25].

This shortcoming is related to the fact that dose accumulation is still lacking in today’s clinical routine, even though it could pave the way towards better understanding of clinical outcomes such as treatment toxicities. After confirming the feasibility [26] and accuracy of contour-based deformable dose accumulation for organs-at-risk (OARs) in prostate MRgRT through phantom validation [27], Bohoudi et al. demonstrated that the total accumulated dose outperformed the baseline dose in predicting acute urinary symptoms for the bladder in ultra-hypofractionated MRgRT [28].

The aim of this study was to investigate the impact of daily online plan adaptation on the accumulated dose for ultra-hypofractionated MRgRT of prostate cancer patients. Therefore, the daily delivered and simulated non-adapted doses for prostate cancer patients were accumulated over all treatment fractions. The baseline plan and the resulting accumulated adapted and non-adapted doses were compared in terms of target coverage and OAR sparing to quantify the benefit of daily plan adaptation.

## 2 Materials and methods

### 2.1 Patient cohort

A total of 23 prostate cancer patients from two institutes (cohort 1: fifteen patients treated at the University Hospital of LMU Munich; cohort 2: eight patients treated at the Heidelberg University Hospital; see Table S1 for details; mean age 67 years; range from 49 years to 84 years) were included in this study. All patients were in the low/intermediate or early high-risk group without distant metastases and participated in the multi-centric *stereotactic MRI-guided radiation therapy for localized prostate cancer* (SMILE) study (ethic project number LMU: 20–291; Heidelberg: S-915/2020). None of the patients had received prior pelvic radiation therapy or local therapy of the prostate gland [13]. All patients received definitive stereotactic MRgRT with a fractionation scheme of 5 × 7.5 Gy at a 0.35 T MRIdian MR-linac (ViewRay Inc., Oakwood Village, OH, USA) [6], [10]. All patients gave informed consent, and the study was approved by the local ethics committees.

### 2.2 Clinical workflow

To ensure consistent bladder and rectum filling, the patients were instructed to follow a drinking and eating protocol for both planning imaging and subsequent irradiation sessions [13], [18]. The non-contrast enhanced planning MRI (pMRI) was acquired at the MR-linac using a clinical balanced steady-state free precession sequence (bSSFP) with an isotropic voxel of size (1.5 mm)^3^ with the patient in supine position (TrueFISP 2D sequence; TR/TE: 3.38 ms/1.45 ms; flip angle: 60°). A planning CT image (pCT; voxel size of 1.0 × 1.0 × 3.0 mm^3^) was acquired using the same patient setup immediately after the pMRI acquisition. Deformable image registration (DIR) of the pCT to the pMRI was automatically performed in the clinical MRIdian treatment planning system (TPS) to create a baseline synthetic CT (sCT) image for dose calculation. Contours were delineated on the pMRI. The CTV included the prostate and, in case of an intermediate risk profile, the base of the seminal glands [13]. The PTV enclosed the CTV with an isotropic margin of 3.0 mm [13], [22], [29]. All patients received step-and-shoot intensity-modulated radiation therapy (IMRT).

Baseline plans with a 6 MV flattening filter-free photon beam were created. These plans were calculated on a (3.0 mm)^3^ dose grid using a Monte Carlo algorithm with a statistical uncertainty of 1% [30]. The aim of the baseline plan was to cover at least 95% of the PTV with 95% of the prescribed dose (35.63 Gy). According to the SMILE protocol, the near-maximum doses (*D*_0.2cc_) to the bladder and rectum should be ≤ 38.5 Gy. For urethral sparing, the avoidance volume defined as the urethra with an isotropic expansion of 2 mm (urethra^+2 mm^) should receive *D*_0.2cc_ ≤ 37.5 Gy.

At every treatment fraction, a daily MRI (dMRI) was acquired using the same sequence as for the pMRI. A daily sCT was generated using pMRI-to-dMRI DIR. The translational patient setup error was corrected using soft tissue alignment, followed by a couch shift [22]. The target structures were rigidly transferred to the dMRI, and OAR structures were deformed to the dMRI using DIR, followed by manual corrections when necessary [7]. For time efficiency, contour corrections were focused on the region within 3 cm of the PTV, which was the area with the highest dose gradients. Online plan adaptation was performed for every fraction using the same objectives as for the baseline plan, and the beam was gated during dose delivery.

For this study, we exported the pMRI, all dMRIs, the daily adapted dose distributions as well as the non-adapted dose distributions, obtained from recalculating the baseline treatment plan on the dMRIs, which were aligned to the pMRI via soft tissue matching in 3D.

### 2.3 Dose accumulation and analysis

For dose accumulation, the pipeline described by Rabe et al. [31] for central lung tumors was implemented and adapted for prostate cancer. In a research version of the TPS RayStation (RaySearch Laboratories, Stockholm, Sweden; research version 10B-R), the hybrid intensity and structure based ANAtomically CONstrained Deformation Algorithm (ANACONDA) [32] was utilized to deformably register the five dMRIs to the pMRI (DIR_1_). Additionally, a solely structure-based DIR approach without considering intensity information was pursued (DIR_2_). Both approaches were separately applied to generate two deformation vector fields (DVFs), and both were by default invertible [32]. For all DIRs, the bladder, rectum, and the intersection of urethra^+2mm^ with the CTV were set as controlling regions of interest (ROIs). This allowed fast convergence during the deformation by penalizing surface distance between the controlling ROIs on pMRI and dMRI in the optimization, even if the differences between both contours were large. Moreover, the union of the bladder, rectum, and CTV, isotropically expanded by 4 cm on the pMRI was chosen as the focus ROI (focus region of the DIR) [33]. All results were visually evaluated using overlay plots. The DIR accuracy was evaluated by calculating the Dice Similarity Coefficient (DSC) and the 95th percentile Hausdorff Distance (HD95) between deformed and planning contours of the controlling ROIs and the CTV using Plastimatch (version 1.8.0) [34].

Subsequently, both non-adapted and adapted fraction doses were mapped to the pMRI using the corresponding DVFs and summed to derive the accumulated non-adapted dose (*D*^non-adapt^) and the accumulated adapted dose (*D*^adapt^) per patient. The baseline plan dose was denoted by *D*^base^. To minimize the impact of the urethra sparing on target coverage analysis, additional target contours were generated on the pMRI: PTV* was defined as PTV excluding the urethra^+2 mm^ and CTV* as CTV excluding the urethra. Thereafter, DVH parameters PTV* *D*_95%_, CTV* *D*_98%_, *D*_50%_, *D*_2%_ and OARs (bladder, rectum, urethra^+2mm^) *D*_0.2cc_ were automatically extracted using Python scripts in the RayStation scripting environment. The relative differences between DVH parameters of *D*^non-adapt^ and *D*^adapt^ normalized to the baseline plan were computed as Δ *D*_x_ = 100% × (*D*_x_^adapt^ - *D*_x_^non-adapt^) / *D*_x_^base^. The differences between *D*_x_^base^ and *D*_x_^adapt^, as well as between *D*_x_^base^ and *D*_x_^non-adapt^ were calculated in a similar manner, respectively. For the comparisons listed above, a two-tailed Wilcoxon signed-rank test was performed with Python (version 3.8.3) using the package Scipy (scipy.stats.wilcoxon; version 1.5.0). Additionally, a Mann-Whitney-*U* test was conducted to compare Δ *D*_x_ of PTV, CTV, and OARs from cohort 1 with these from cohort 2. A statistically significant result was determined by a p-value < 0.05.

## 3 Results

### 3.1 DIR accuracy

The median DSC (interquartile range, IQR, [25%, 75%]) for DIR_1_ and DIR_2_ for the CTV was 0.90 ([0.89, 0.92]) and 0.89 ([0.85, 0.91]), respectively. For the three controlling ROIs bladder, rectum, and urethra^+2mm^ inside the CTV, the median DSC results were between 0.85 and 0.97. For all four contours, the median HD95 was below 4 mm, close to the dose grid size of 3 mm (see Supplementary Materials Table S2). On average, both DIR approaches led to DVH parameters differing by less than 0.2% with respect to *D*^base^. In the following, only results from DIR_1_ are presented.

### 3.2 Accumulated doses

**Fig. 1:**
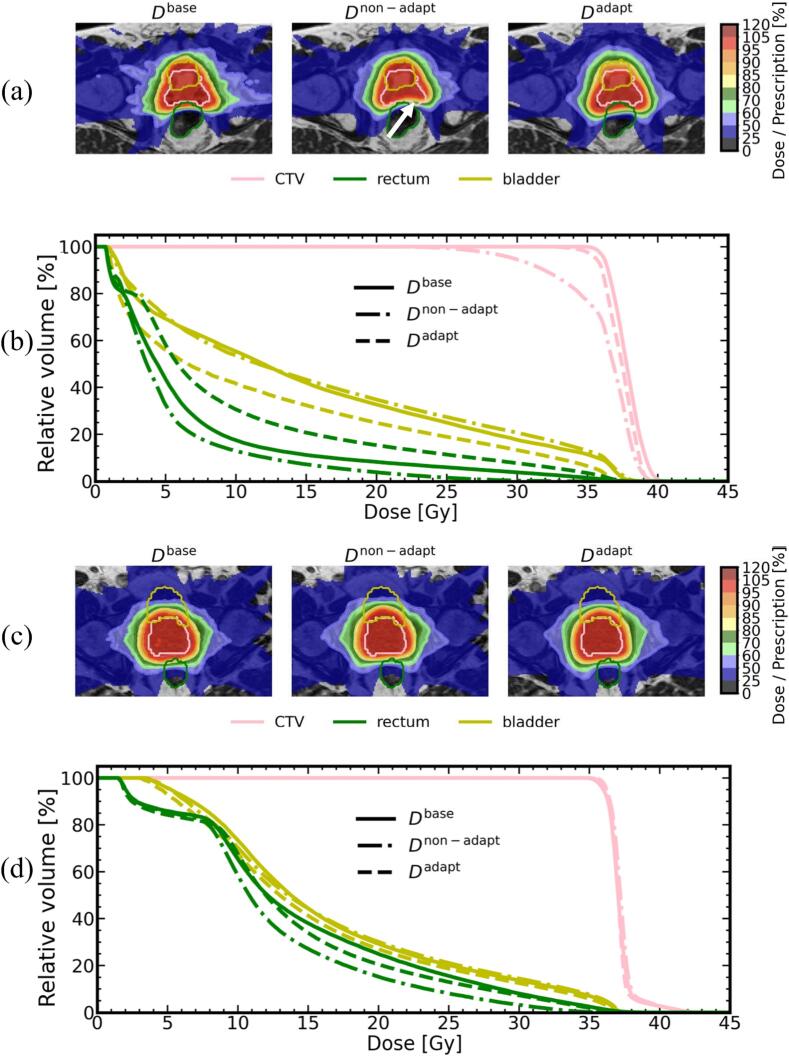
Example accumulated *D*^base^, *D*^non-adapt^, and *D*^adapt^ dose distributions. (a) patient 1 and (c) patient 11 from cohort 1, superimposed on the pMRI, with corresponding DVH curves (b: patient 1, d: patient 11). The white arrow in (a) indicates the area with insufficient CTV coverage. Contours of CTV* (pink), rectum (green) and bladder (yellow) are displayed. All results from DIR_1_-based accumulation.

Exemplary accumulated dose distributions and DVHs of *D*^base^, *D*^non-adapt^, and *D*^adapt^ for two patients are depicted in Fig. 1. In one case (patient 1), the patient underwent significant inter-fractional bladder volume changes (range of volumes: [118, 413] cm^3^) during the treatment, resulting in large differences among the three dose distributions. The target coverage was degraded in the soft tissue matching scenario and was clearly improved after the adaptation. Conversely, the other patient case (patient 11), exhibited similar DVHs for the three dose distributions.

**Fig. 2:**
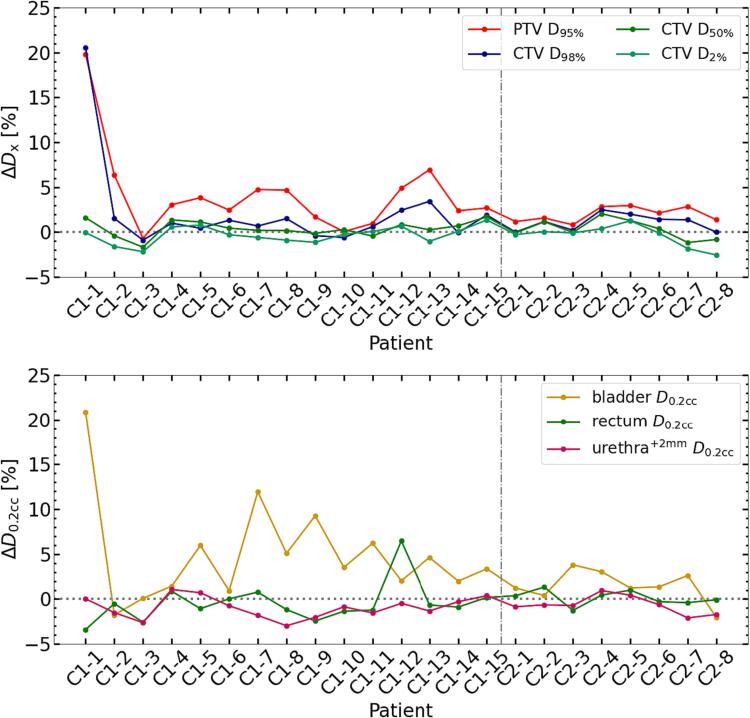
Per patient results. Top row: Δ*D*_95%_ for PTV* and Δ*D*_98%,_Δ*D*_50%,_Δ*D*_2%_ for CTV*. Bottom row: Δ*D*_0.2cc_ for bladder, rectum, and urethra^+2mm^. Cohort 1 patients are labelled with C1, and cohort 2 patients with C2. Data points are connected for improved visibility. The vertical grey line separates the cohorts.

**Fig. 3:**
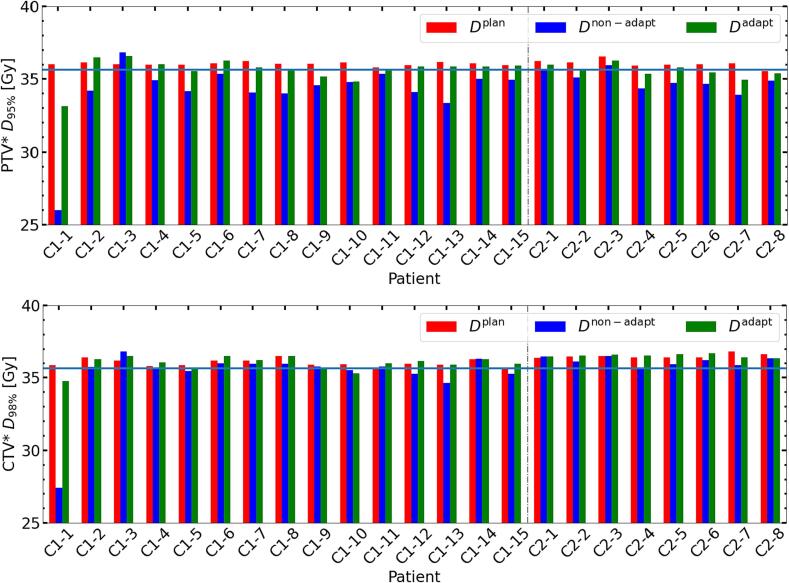
Patient-specific DVH parameters compared with 95% of the prescription (blue horizontal line). Top row: PTV* *D*_95%_ derived from *D*^base^ (red), *D*^non-adapt^ (blue), and *D*^adapt^ (green). Bottom row: CTV* *D*_98%_ for the same three doses. C1 stands for cohort 1, C2 stands for cohort 2. Y-axis is cropped for better visibility. The vertical grey line separates the cohorts.

**Fig. 4:**
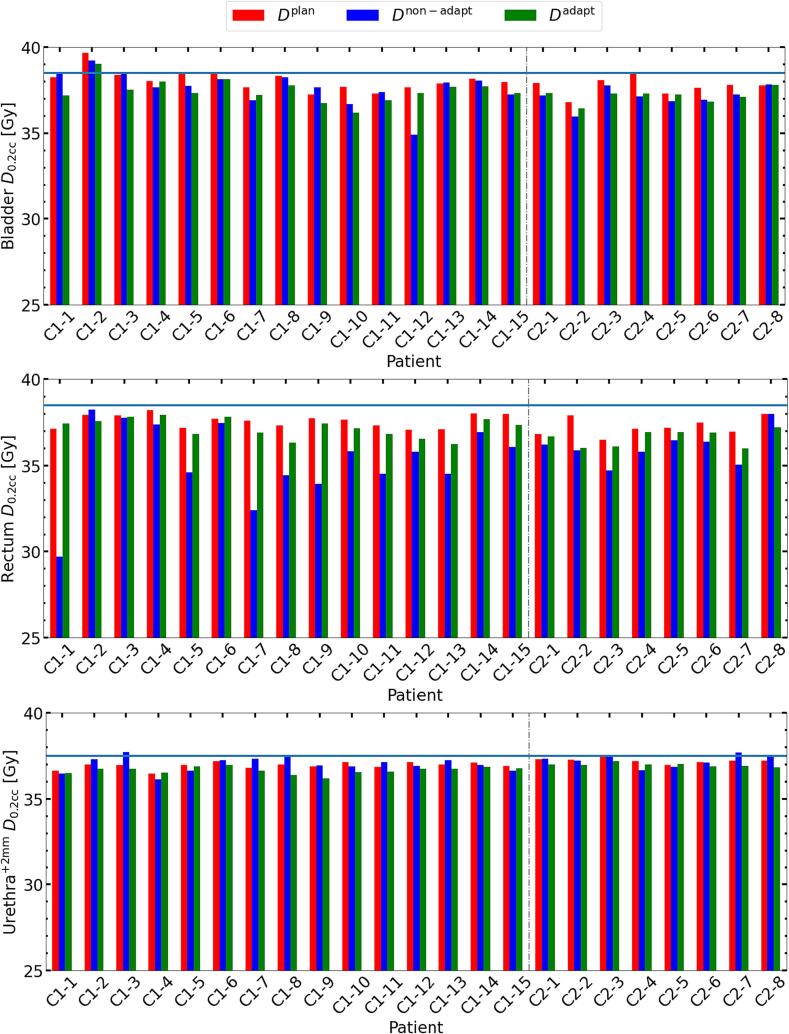
Patient-specific *D*_0.2cc_ of the OARs. Top row: bladder. Middle row: rectum. Lower row: urethra^+2mm^. These were compared with their respective constraints (blue horizontal line). *D*^base^ (red), *D*^non-adapt^ (blue), and *D*^adapt^ (green) are shown next to each other. C1 stands for cohort 1, C2 stands for cohort 2. Y-axis is cropped for better visibility. The vertical grey line separates the cohorts.

The median Δ *D*_x_ (IQR) over all patients, along with the average difference between *D*_x_^base^ and *D*_x_^adapt^, and *D*_x_^base^ and *D*_x_^non-adapt^ are summarized in Table 1. Adaptation led to increased PTV* *D*_95%_ by 2.7% ([1.5, 4.3]%) and CTV* *D*_98%_ by 1.2% ([0.1, 1.7]%) compared to no adaptation, both differences were statistically significant. For bladder and urethra^+2mm^, *D*_0.2cc_ decreased by 0.4% ([−1.2, 0.4]%) and 0.8% ([−1.6, 0.1]%), respectively, and the urethra^+2mm^ *D*_0.2cc_ difference was significant. For rectum, *D*_0.2cc_^adapt^ was significantly higher by 2.6% ([1.2, 4.9]%) than *D*_0.2cc_^non-adapt^, and both values were below *D*_0.2cc_^base^ for most patients. Patient-per-patient Δ *D*_x_ for targets and OARs are shown in Fig. 2. Results of the Mann-Whitney-*U* test showed that none of the differences was significant. Patient 1 (see Fig. 1) had the two most elevated Δ *D*_x_ values as shown in upper panel of Fig. 2. Except from one patient of cohort 1 (patient 3), PTV* *D*_95%_ increased through adaptation. For rectum Δ *D*_0.2cc_, values were positive for most patients (21/23), while these were negative for bladder and urethra^+2mm^ for fourteen and seventeen patients from both cohorts, respectively. Compared to cohort 2, cohort 1 exhibited a tendency towards higher rectum Δ *D*_0.2cc_ and corresponding higher PTV* Δ *D*_95%_. In Fig. 3, the PTV* *D*_95%_ for *D*^base^, *D*^non-adapt^, and *D*^adapt^ is shown to verify that coverage satisfied 95% of the prescribed dose. We also evaluated the CTV* *D*_98%_ with the same condition. For all patients except from one of cohort 1 (patient 3), the adaptation yielded accumulated doses closer to PTV* *D*_95%_^base^. No significant difference between the CTV* *D*_98%_^adapt^ and *D*_98%_^base^ was observed (see Table 1). Among the 23 patients, seven patients did not meet the PTV* *D*_95%_^adapt^ planning objective, but only two of them had an inadequate CTV* *D*_98%_^adapt^. In case of no adaptation, 20 out of 23 patients had insufficient PTV* *D*_95%_^non-adapt^ and six of them had inadequate CTV* *D*_98%_^non-adapt^. Fig. 4 shows the *D*_0.2cc_ for OARs and their respective dose constraints. Except for the bladder *D*_0.2cc_^adapt^ of patient 2 from cohort 1, *D*_0.2cc_^adapt^ for all other patients were within the constraints. Although Fig. 2 indicates an increase in rectum near-maximum doses, these remained below the constraint. *D*_0.2cc_ for the urethra^+2mm^ in the *D*^non-adapt^ was exceeding or at the limit for five patients, while through the adaptation, this was avoided.

**Table 1:** Median values (IQR [25 %, 75 %]) of Δ*D*_x_, *D*_x_^base^ versus *D*_x_^non-adapt^, and *D*_x_^base^ versus *D*_x_^adapt^. Significance of differences (*p* < 0.05) is indicated by a dagger (†). All values are given in percent. The results over all patients are shown in a). The results from both cohorts are individually presented in b) and c).

a) Both cohorts							
	PTV* D_95%_	CTV* *D*_98%_	CTV* *D*_50%_	CTV* D_2%_	Bladder *D*_0.2cc_	Rectum *D*_0.2cc_	Urethra^+2mm^*D*_0.2cc_
Δ*D*_x_	2.7† [1.5, 4.3]	1.2† [0.1, 1.7]	0.3 [−0.1, 1.2]	−0.1 [−1.0, 0.2]	−0.4 [−1.2, 0.4]	2.6† [1.2, 4.9]	−0.8† [−1.6, −0.1]
$\frac{D_{x}^{non-adapt}-D_{x}^{base}}{D_{x}^{base}}$	−3.7† [−5.3, −2.4]	−0.9† [−1.7, −0.3]	−0.3† [−1.0, 0.0]	−0.8† [−1.3, −0.6]	−1.1† [−1.9, 0.0]	−4.8† [−6.9, −2.0]	−0.1 [−0.5, 0.7]
$\frac{D_{x}^{adapt}-D_{x}^{base}}{D_{x}^{base}}$	−0.8† [−1.4, −0.3]	0.3 [−0.5, 0.7]	0.0 [−0.4, 0.1]	−1.1† [−1.8, −0.7]	−1.4† [−2.1, −0.9]	−1.0† [−1.7, −0.7]	−0.7† [−0.8, −0.5]

b) Cohort 1							
	PTV* D_95%_	CTV* *D*_98%_	CTV* *D*_50%_	CTV* D_2%_	Bladder *D*_0.2cc_	Rectum *D*_0.2cc_	Urethra^+2mm^*D*_0.2cc_
Δ*D*_x_	3.1† [2.0, 4.8]	1.0† [0.2, 1.7]	0.3 [0.0, 1.0]	−0.2 [−1.0, 0.3]	−0.9 [−1.3, 0.1]	3.5† [1.7, 6.1]	−0.9† [−1.7, −0.1]
$\frac{D_{x}^{non-adapt}-D_{x}^{base}}{D_{x}^{base}}$	−4.1† [−5.5, −2.8]	−1.0† [−1.7, −0.3]	−0.5†[−0.9, 0.0]	−0.8† [−1.3, −0.6]	−0.9† [−1.9, 0.1]	−5.0† [−7.6, −2.5]	0.1 [−0.6, 0.8]
$\frac{D_{x}^{adapt}-D_{x}^{base}}{D_{x}^{base}}$	−0.6 [−1.1, 0.0]	0.0 [−0.5, 0.8]	0.0 [−0.4, 0.1]	−1.2† [−1.8, −0.7]	−1.3† [−2.0, −1.0]	−1.0† [−1.6, −0.8]	−0.7† [−0.9, −0.4]

c) Cohort 2							
	PTV* D_95%_	CTV* *D*_98%_	CTV* *D*_50%_	CTV* D_2%_	Bladder *D*_0.2cc_	Rectum *D*_0.2cc_	Urethra^+2mm^*D*_0.2cc_
Δ*D*_x_	1.9† [1.3, 2.8]	1.3† [0.2, 1.6]	0.2 [−0.3, 1.2]	−0.1 [−0.6, 0.1]	0.1 [−0.3, 0.6]	1.3 [1.0, 2.7]	−0.7 [−1.1, −0.4]
$\frac{D_{x}^{non-adapt}-D_{x}^{base}}{D_{x}^{base}}$	−3.2† [−3.9, −1.8]	−0.8† [−1.5, −0.4]	−0.2† [−1.0, −0.1]	−0.8† [−1.1, −0.5]	−1.7† [−2.0, −1.1]	−3.3† [−4.9, −1.8]	−0.1 [−0.2, 0.2]
$\frac{D_{x}^{adapt}-D_{x}^{base}}{D_{x}^{base}}$	−1.1† [−1.6, −0.6]	0.3 [0.0, 0.5]	0.1 [−0.4, 0.2]	−1.0† [−1.5, −0.8]	−1.7† [−2.1, −0.8]	−1.3† [−2.1, −0.6]	−0.8† [−0.8, −0.6]

## 4 Discussion

To assess the impact of daily online plan adaptation in ultra-hypofractionated MRgRT for prostate cancer patients, we compared accumulated adapted doses to simulated non-adapted doses on pMRI for 23 patients from two institutes in terms of target coverage and sparing of OARs. For both cohorts, the PTV* *D*_95%_ and CTV* *D*_98%_ increased significantly by 2.7% ([1.5, 4.3]%) and 1.2% ([0.1, 1.7]%), by daily adaptation. Without adaptation, the PTV* *D*_95%_ would have been significantly lower than planned by 3.7% ([−5.3, −2.4]%). Through adaptation, no significant differences of CTV* *D*_98%_ and *D*_50%_ to the baseline plan were observed anymore. Adaptation thus ensured that the planned target coverage was more closely recovered in presence of inter-fractional changes. For the OARs, online adaptation led to a reduction of *D*_0.2cc_ in bladder (not significant) and urethra^+2mm^ (significant), while rectum *D*_0.2cc_ increased significantly. Following adaptation, except for a single case, all the OARs *D*_0.2cc_ met the protocol-defined dose constraints. Especially, the violation of urethra^+2mm^ *D*_0.2cc_ constraint was avoided in five patients through online adaptation. While the urethra^+2mm^ *D*_0.2cc_^adapt^ was significantly lower than *D*_0.2cc_^base^, the *D*_0.2cc_^non-adapt^ was not. It is likely that the rectum *D*_0.2cc_ increase within the constraint was necessary to ensure target coverage. These results highlight the potential benefits of online adaptation in improving target coverage at the level of the accumulated dose. Particularly in presence of strong inter-fractional anatomical changes, adaptation was found to play a crucial role in achieving adequate target coverage, while the OAR sparing was still maintained (see patient 1 in Fig. 1 for example). On the level of individual fractions, Nierer et al. concurringly reported that in single fractions, adaptation was crucial to ensure target coverage. It was, however, also pointed out that there was on average no significant change of OAR exposure resulting from online adaptation [22]. The phase three randomized clinical trial conducted by Kishan et al. demonstrated the superiority of MRgRT compared to CT-guided radiotherapy for prostate cancer in effectively reducing both moderate acute physician-scored toxic effects and decrements in patient-reported quality of life [35]. In fact, online adaptation could result in more pronounced benefits in OAR sparing for certain indications, such as lung, liver, and adrenal metastases [36], [37], [38], [39], [40].

For dose accumulation, DIR accuracy is critical. We investigated two different approaches, both of which achieved average DSCs above 0.80 for all structures of interest, as recommended by American Association of Physicists in Medicine (AAPM) TG 132 [41]. Ultimately, both DIRs produced similar accumulated DVH parameters (mean deviation of 0.2%), confirming the robustness of the used DIR settings and accumulated dose distributions.

The degree of improvement in target coverage and OARs protection varied depending on the cohort. Due to physician choices, cohort 1 had in contrast to cohort 2 partially higher rectum doses after adaptation, while the magnitude of PTV* *D*_95%_ improvement was also larger. However, these differences were not significant. Several factors contribute to the differences in rectum Δ *D*_0.2cc_ and PTV* Δ *D*_95%_ observed between both cohorts. Firstly, variations in plan robustness, influenced by differing conformity of the high dose region and the use of different cost functions during dose optimization, were observed. Secondly, differences in patient preparation, notably in drinking protocols (cohort 1 was advised to drink 750 mL of water, cohort 2 only 250 mL), could contribute to different organ dynamics during the treatment course. In general, it is expected that interfractional anatomic changes can potentially blur the steep falloffs of doses across the fractions during accumulation, which could also lead to reduced near maximum DVH parameter, like *D*_0.2cc._

Owing to the small number of treatment fraction in the ultra-hypofractionated treatment scheme, the impact of adaptation at each fraction could be stronger compared to normo- or hypo-fractionated treatments, increasing the importance of verifying the accumulated dose. In particular, dose accumulation allows monitoring the delivered dose and comparing it to the initially planned dose.

Generally, the dose to the urethra^+2mm^ needs to be considered with care. Despite the higher soft tissue contrast of MRI, the exact delineation of the urethra was challenging. Moreover, the small volume of the urethra^+2mm^ made it more susceptible to the statistical uncertainties of Monte Carlo dose calculations and DIR errors. To mitigate this, the urethra^+2mm^ intersection with the CTV was used as a controlling ROI during the DIR. This aimed to minimize the impact of dose interpolation and uncertainties in dose accumulation in this region.

The pipeline implemented for dose accumulation in prostate cancer MRgRT might in the future also support the analysis of correlations between total accumulated dose and the acute as well as late toxicities in different OARs [42]. With the introduction of deep learning-based auto segmentation of OARs into clinics, additional contouring of neurovascular structures such as the penile bulb and internal pudendal arteries becomes notably more time efficient [43], [44], [45], [46], [47]. To assess the clinically relevant impact of daily plan adaptation on these additional structures, especially in treatment schemes focusing on neurovascular sparing, similar methodology as used for this study can be leveraged.

Moreover, the outcomes of this study offer valuable insights applicable to other online adaptive image-guided radiation therapy techniques, such as CBCT-guided radiation therapy [48].

In conclusion, online adaptation in MRgRT was found to be advantageous in improving target coverage and OARs sparing, especially for patients experiencing strong anatomical changes. However, the average improvement was limited for most patients.

## CRediT authorship contribution statement

**Yuqing Xiong:** Investigation, Software, Formal analysis, Data curation, Writing – original draft, Visualization. **Moritz Rabe:** Conceptualization, Writing – review & editing, Supervision. **Carolin Rippke:** Data curation, Writing – review & editing. **Maria Kawula:** Data curation, Writing – review & editing. **Lukas Nierer:** Data curation, Writing – review & editing. **Sebastian Klüter:** Writing – review & editing, Supervision. **Claus Belka:** Writing – review & editing, Supervision. **Maximilian Niyazi:** Writing – review & editing, Supervision. **Juliane Hörner-Rieber:** Writing – review & editing, Supervision. **Stefanie Corradini:** Writing – review & editing, Supervision. **Guillaume Landry:** Conceptualization, Writing – review & editing, Supervision. **Christopher Kurz:** Conceptualization, Writing – review & editing, Supervision, Funding acquisition.

## Declaration of Competing Interest

The authors declare the following financial interests/personal relationships which may be considered as potential competing interests: The authors declare that they have no known competing financial interests or personal relationships that could have appeared to influence the work reported in this paper.

## Data Availability

The participants of this study did not give written consent for their data to be shared publicly, so due to the sensitive nature of the research supporting data is not available.
