# Supplementary material for: Impact of daily plan adaptation on accumulated doses in ultra-hypofractionated magnetic resonance-guided radiation therapy of prostate cancer
Source: Phys Imaging Radiat Oncol. 2024 Feb 25;29:100562. doi: 10.1016/j.phro.2024.100562 (PMC10924058; doi:10.1016/j.phro.2024.100562)
Supplement: Supplementary data 1 [file mmc1.docx]

# Supplementary Materials

Table S1: Patient information: age, TNM (tumor, lymph node, metastases) stage, Gleason score, and the size of CTV.

| Patient | | Age | TNM stage | Gleason Score | CTV size [cm^3^] |
| --- | --- | --- | --- | --- | --- |
| Cohort 1 | 1 | 78 | cT1b, cN0, cM0 | 7b | 34.2 |
|  | 2 | 64 | cT1c, cN0, cM0 | 7b | 69.7 |
|  | 3 | 65 | cT1c, cN0, cMx | 7a | 66.3 |
|  | 4 | 72 | cT2b, cN0, cM0 | 6 | 37.8 |
|  | 5 | 59 | cT1c, cN0, cM0 | 6 | 87.5 |
|  | 6 | 62 | cT2c, cN0, cM0 | 7b | 56.5 |
|  | 7 | 59 | cT2c, cN0, cMx | 6 | 79.9 |
|  | 8 | 84 | cT2c, cN0, cM0 | 7a | 63.3 |
|  | 9 | 58 | cT1c, cN0, cM0 | 6 | 54.5 |
|  | 10 | 78 | cT1c, cN0, cMx | 7a | 70.0 |
|  | 11 | 64 | cT1c, cN0, cM0 | 7a | 63.7 |
|  | 12 | 74 | cT1, cN0, cMx | 7a | 63.5 |
|  | 13 | 66 | cT1c, cN0, cMx | 7a | 47.0 |
|  | 14 | 66 | cT1c, cN0, cMx | 6 | 40.1 |
|  | 15 | 78 | cT1, cN0, cM0 | 7a | 39.9 |
| Cohort 2 | 1 | 78 | cT2a, cN0, cM0 | 7a | 57.2 |
|  | 2 | 63 | cT2a, cN0, cM0 | 7a | 76.4 |
|  | 3 | 64 | cT1c, cNx, cMx | 6 | 71.0 |
|  | 4 | 68 | cT1c, cN0, cM0 | 7a | 78.9 |
|  | 5 | 68 | cT1c, cN0, cMx | 6 | 81.0 |
|  | 6 | 49 | cT2, cN0, cMx | 7a | 40.5 |
|  | 7 | 64 | cT1c, cN0, cM0 | 6 | 64.5 |
|  | 8 | 68 | cT2c, cN0, cMx | 7a | 86.7 |

Table S2: Median values (IQR [25%, 75%] in brackets) of DSC and HD95 for DIR_1_ and DIR_2_ for CTV, bladder, rectum, and urethra^+2mm^-CTV-intersection.

| [%] \| [mm] | CTV | | Bladder | | Rectum | | Urethra^+2mm^-CTV-intersection | | | |
| --- | --- | --- | --- | --- | --- | --- | --- | --- | --- | --- |
|  | DSC | HD95 | DSC | HD95 | DSC | HD95 | | DSC | HD95 |  |
| DIR_1_ | 0.90  [0.89, 0.92] | 3.25  [2.56, 3.62] | 0.96  [0.96, 0.97] | 1.67  [1.50, 1.81] | 0.93  [0.92, 0.94] | 1.56  [1.50, 2.12] | | 0.85  [0.83, 0.88] | 1.50  [1.50, 1.51] |  |
| DIR_2_ | 0.89  [0.86, 0.91] | 3.77  [3.00, 4.41] | 0.97  [0.96, 0.97] | 1.50  [1.50, 2.36] | 0.94  [0.93, 0.95] | 1.50  [1.50, 1.68] | | 0.86  [0.84, 0.99] | 1.50  [1.50, 1.51] |  |
